# The Role of Nuclear Bodies in Gene Expression and Disease

**DOI:** 10.3390/biology2030976

**Published:** 2013-07-09

**Authors:** Marie Morimoto, Cornelius F. Boerkoel

**Affiliations:** Department of Medical Genetics, Child & Family Research Institute, University of British Columbia, Vancouver, BC V5Z 4H4, Canada; E-Mail: mmorimoto@cfri.ca

**Keywords:** nuclear bodies, transcription, gene expression, genome organization

## Abstract

This review summarizes the current understanding of the role of nuclear bodies in regulating gene expression. The compartmentalization of cellular processes, such as ribosome biogenesis, RNA processing, cellular response to stress, transcription, modification and assembly of spliceosomal snRNPs, histone gene synthesis and nuclear RNA retention, has significant implications for gene regulation. These functional nuclear domains include the nucleolus, nuclear speckle, nuclear stress body, transcription factory, Cajal body, Gemini of Cajal body, histone locus body and paraspeckle. We herein review the roles of nuclear bodies in regulating gene expression and their relation to human health and disease.

## 1. Introduction

Gene expression is a multistep process that is vital for the development, adaptation and survival of all living organisms. Regulation of gene expression occurs at the level of transcription, RNA processing, RNA export, translation and protein degradation [1,2,3]. The nucleus has the ability to modulate gene expression at each of these levels. How the nucleus executes this regulation is gradually being dissected. Of particular interest for this review is the spatial relationship between genes and the proteins and non-coding RNAs that regulate their expression. Specifically, are the regulatory components randomly dispersed throughout the nucleus or concentrated within regions?

**Figure 1:**
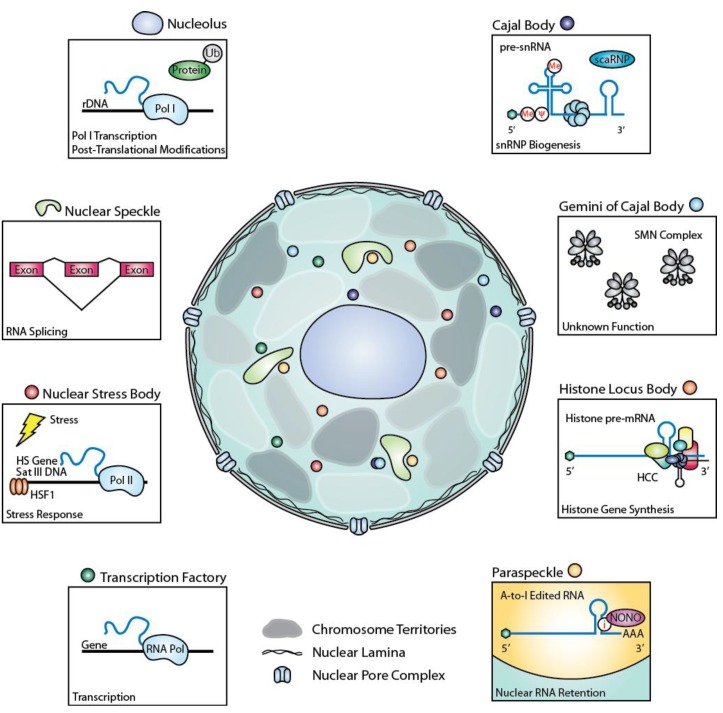
Diagram summarizing the role of nuclear bodies in gene expression.Abbreviations: A-to-I; adenosine to inosine; HCC, histone cleavage complex; HS, heat shock; HSF1, heat shock factor 1; i, inosine; Me, 2'-O-methylation; Pol, RNA polymerase; pre-mRNA, precursor messenger RNA; pre-snRNA, precursor small nuclear RNA; rDNA, ribosomal DNA; Sat III DNA, satellite III DNA; scaRNP, small Cajal body-specific ribonucleoprotein; SMN, survival of motor neuron complex; snRNP, small nuclear ribonucleoprotein; Ub, ubiquitin; Ψ, pseudouridylation.

Part of the answer lies in the compartmentalization of the nuclear space into nuclear bodies of specific functions. Nuclear bodies are broadly defined as morphologically distinct regions within the nucleus; they are distinguishable from their surroundings by techniques, such as transmission electron microscopy, differential interference contrast microscopy and immunofluorescent detection of proteins that localize to a nuclear body of interest. Similar to conventional cytoplasmic organelles, nuclear bodies are distinct local environments of unique functions (Figure 1). Nuclear bodies include the nucleolus, the nuclear speckle, the nuclear stress body, the transcription factory, the Cajal body, the Gemini of Cajal body, the histone locus body and the paraspeckle. Immunofluorescent images of these nuclear bodies can be viewed in the Nuclear Protein Database (http://npd.hgu.mrc.ac.uk). The diverse mechanisms of gene regulation associated with nuclear bodies add another dimension to our understanding of gene regulation. 

## 2. Nuclear Bodies and Mechanisms of Gene Expression

In this review, we focus on the role of nuclear bodies in gene regulation and discuss human diseases associated with mutations in genes encoding for key components. A review of the perinucleolar compartment is deferred, since it has so far only been identified in cancerous cells, and its relevance to general biology remains unclear. Additionally, although the promyelocytic leukemia (PML) body, the clastosome, the cleavage body, the Oct1/PTF/transcription (OPT) domain, the polymorphic interphase karyosomal association (PIKA) domain and the polycomb group (PcG) body contribute to the regulation of gene expression [4,5], these are not reviewed, because of the limited available information.

### 2.1. Nucleolus

#### 2.1.1. Discovery

The nucleolus is one of the most prominent and clearly visible structures in the nucleus. Described by the anatomist Rudolf Wagner in 1835 and by the physiologist Gabriel Valentin the following year, the nucleolus has fascinated scientists for decades [6,7]. Though initially characterized as the major center for ribosome biogenesis [8], it has become increasingly apparent that the nucleolus is a multifunctional nuclear body that also participates in mitosis, cell cycle regulation, DNA replication, DNA repair, ribonucleoprotein (RNP) biogenesis and the stress response [9,10,11].

#### 2.1.2. Key Components

The nucleolus is composed of a fibrillar center surrounded by dense fibrillar and granular components. Among the several hundreds of proteins found in the nucleolus, there are proteins for ribosomal biogenesis, chromatin structure and messenger RNA (mRNA) metabolism, as well as ribosomal proteins, chaperones, translation factors and many others of unknown function [12,13]. These proteins include (1) the phosphoproteins, nucleophosmin and nucleolin, (2) the core members of the box C/D small nucleolar RNP (snoRNP) complex (fibrillarin, NHP2-like protein 1 (NHP2L1), nucleolar protein 56 (NOP56) and nucleolar protein 58 (NOP58)), (3) the core members of the H/ACA snoRNP complex (dyskerin, GAR1, NHP2 and nucleolar protein 10 (NOP10)), (4) the protein chaperone nucleolar phosphoprotein 140 kDa (NOPP140), (5) RNA Polymerase I (RNA Pol I), and (6) proteins of the 40S and 60S ribosomal subunits (Table 1). Approximately 700 nucleolar proteins have now been identified and catalogued in the Nucleolar Proteome Database (http://www.lamondlab.com/NOPdb/). RNA members include the box C/D and H/ACA small nucleolar RNAs (snoRNAs); these are incorporated into the snoRNPs that process precursor ribosomal RNA (pre-rRNA) (Table 1).

#### 2.1.3. Functions of Key Components

Most proteins in the nucleolus participate in ribosomal RNA (rRNA) transcription and pre-rRNA processing [12,13] (Table 1). Nucleophosmin is a multifunctional chaperone involved in ribosome biogenesis and transport [14], and nucleolin is involved in RNA Pol I transcription and pre-rRNA processing [15,16]. The box C/D and H/ACA snoRNP complexes process pre-rRNA through the post-transcriptional modifications, 2'-O-methylation and pseudouridylation, respectively [17]. NOPP140 likely functions as a chaperone that shuttles the snoRNPs between the Cajal bodies and nucleoli [18]. The 18S, 5.8S and 28S rRNAs, transcribed by RNA Pol I, and the ribosomal proteins constitute the ribosome. Defects of rRNA transcription and rRNA processing lead to reduced ribosome biogenesis and protein synthesis (Table 2).

**Table 1:** Summary of the key components of nuclear bodies.

Nuclear Body Component	Synonyms	Notes	Nuclear Body Component Localization References
**Nucleolus**			
Proteins			
Nucleophosmin	B23	Multifunctional chaperone	[19]
Nucleolin	C23	pre-rRNA processing RNA polymerase I transcription	[19]
Fibrillarin		Box C/D snoRNP component pre-rRNA 2'-O-methyltransferase	[20]
NHP2L1	15.5K NHPX	Box C/D snoRNP component pre-rRNA 2'-O-methylation	[21]
NOP56		Box C/D snoRNP component pre-rRNA 2'-O-methylation	[22]
NOP58		Box C/D snoRNP component pre-rRNA 2'-O-methylation	[22]
Dyskerin	DKC1 NAP57 NOLA4	H/ACA snoRNP component pre-rRNA pseudouridylase Telomerase component	[23]
GAR1	NOLA1	H/ACA snoRNP component pre-rRNA pseudouridylation Telomerase component	[24]
NHP2	NOLA2	H/ACA snoRNP component pre-rRNA pseudouridylation Telomerase component	[25]
NOP10	NOLA3	H/ACA snoRNP component pre-rRNA pseudouridylation Telomerase component	[25]
NOPP140	NOLC1	Box C/D snoRNP chaperone Transcription	[26]
RNA polymerase I		Transcription	[12,13,27]
40S ribosomal subunit		40S small ribosomal subunit Translation	[12,13]
60S ribosomal subunit		60S large ribosomal subunit Translation	[12,13]
**Nucleolus**			
RNAs			
*U3* snoRNA		Box C/D snoRNA pre-rRNA processing	[28]
*U8* snoRNA		Box C/D snoRNA pre-rRNA processing	[28,29]
*U13* snoRNA		Box C/D snoRNA pre-rRNA processing	[28]
*U14* snoRNA		Box C/D snoRNA pre-rRNA processing	[29]
*U17* snoRNA	*E1* snoRNA	Box H/ACA snoRNA pre-rRNA processing	[30]
*E2* snoRNA		Box H/ACA snoRNA pre-rRNA processing	[30]
*E3* snoRNA		Box H/ACA snoRNA pre-rRNA processing	[30]
**Nuclear Speckle**			
Proteins			
SRSF1	SF2 ASF SRp30a	SRSF family Constitutive and alternative splicing	[31]
SRSF2	SC35 SRp30b	SRSF family Constitutive and alternative splicing	[32]
SRSF3	SRp20	SRSF family Constitutive and alternative splicing	[31]
SRSF4	SRp75	SRSF family Constitutive and alternative splicing	[33,34]
SRSF5	SRp40	SRSF family Constitutive and alternative splicing	[34]
SRSF6	SRp55	SRSF family Constitutive and alternative splicing	[33,34]
SRSF7	9G8	SRSF family Constitutive and alternative splicing	[34]
CLK	STY	Dual specificity protein kinase SRSF phosphorylation	[35]
PRP3		U4/U6 snRNP component pre-mRNA splicing	[34]
PRP6		U4/U6-U5 tri-snRNP component pre-mRNA splicing	[34]
PRP8		U5 snRNP component pre-mRNA splicing	[34]
U5 snRNP 200 kDa protein	BRR2	U5 snRNP component pre-mRNA splicing	[34]
U5 snRNP 116 kDa break/> protein	SNU114	U5 snRNP component pre-mRNA splicing	[33,34]
CPSF160	CPSF1	CPSF complex pre-mRNA 3' end processing	[34]
CPSF100	CPSF2	CPSF complex pre-mRNA 3' end processing	[34]
CPSF73	CPSF3	CPSF complex pre-mRNA 3' end processing	[34]
CPSF30	CPSF4	CPSF complex pre-mRNA 3' end processing	[34]
CFIm68	CPSF6	CFI_m_ complex pre-mRNA 3' end processing	[34]
CSTF64	CSTF2	CSTF complex pre-mRNA 3' end processing	[34]
RNA polymerase II		Transcription	[34,36]
SON		pre-mRNA splicing	[34]
MAGOH		Exon junction complex mRNA export and NMD	[34]
eIF4AIII	DDX48	Exon junction complex mRNA export and NMD	[34]
RNPS1		Exon junction complex mRNA export and NMD	[33,34]
Y14	RBM8A	Exon junction complex mRNA export and NMD	[34]
Aly/REF		Exon junction complex mRNA export and NMD	[34]
RAE1		mRNA export	[34]
RNAs			
*U1* snRNA		U1 snRNP component pre-mRNA splicing	[37]
*U2* snRNA		U2 snRNP component pre-mRNA splicing	[37]
*MALAT1* RNA	*NEAT2*	Long ncRNA	[38]
Poly(A)+ RNA		RNA with a poly(A) tail	[39]
**Nuclear Stress Body**			
Proteins			
HSF1		Heat shock transcription factor family Transcription	[40]
HSF2		Heat shock transcription factor family Transcription	[41]
SAF-B	HAP HET	S/MAR binding protein Transcription	[42]
Sam68	KHDRBS1	Transcription Alternative splicing	[43]
SRSF1	ASF SF2	SRSF family Constitutive and alternative splicing	[43]
SRSF7	9G8	SRSF family Constitutive and alternative splicing	[43]
SRSF9	SRp30c	SRSF family Constitutive and alternative splicing	[43]
RNA polymerase II		Transcription	[44]
RNA			
Satellite III ncRNA		Long ncRNA transcribed from the pericentric heterochromatic 9p12 locus	[44]
**Transcription Factory**			
Proteins			
RNA polymerase I		Transcription	[45,46]
RNA polymerase II		Transcription	[45,46]
RNA polymerase III		Transcription	[45,46]
**Cajal Body**			
Proteins			
Coilin		Function unknown	[47,48]
Fibrillarin		Box C/D snoRNP component pre-rRNA 2'-O-methyltransferase	[49]
NHP2L1	15.5K NHPX	Box C/D snoRNP component pre-rRNA 2'-O-methylation	[21,50]
NOP56		Box C/D snoRNP component pre-rRNA 2'-O-methylation	[50]
NOP58		Box C/D snoRNP component pre-rRNA 2'-O-methylation	[50]
Dyskerin	DKC1 NAP57 NOLA4	H/ACA snoRNP component pre-rRNA pseudouridylase Telomerase component	[51]
GAR1	NOLA1	H/ACA snoRNP component pre-rRNA pseudouridylation Telomerase component	[52]
NHP2	NOLA2	H/ACA snoRNP component pre-rRNA pseudouridylation Telomerase component	[52]
NOP10	NOLA3	H/ACA snoRNP component pre-rRNA pseudouridylation Telomerase component	[52]
TERT		Telomerase component Reverse transcriptase	[53]
TCAB1	WRAP53	Telomerase component	[54]
NOPP140	NOLC1	Box C/D snoRNP chaperone Transcription	[18]
SMN1	GEMIN1	SMN complex Spliceosomal snRNP biogenesis	[55]
RNAs			
*U85* scaRNA		scaRNA snRNA modification	[56]
*U87* scaRNA		scaRNA snRNA modification	[56]
*U88* scaRNA		scaRNA snRNA modification	[56]
*U89* scaRNA		scaRNA snRNA modification	[56]
*U90* scaRNA		scaRNA snRNA modification	[56]
*U91* scaRNA		scaRNA snRNA modification	[56]
*U92* scaRNA		scaRNA snRNA modification	[56]
*U2* snRNA		U2 snRNP component pre-mRNA splicing	[57,58]
*U4* snRNA		U4 snRNP component pre-mRNA splicing	[57,58]
*U5* snRNA		U5 snRNP component pre-mRNA splicing	[57,58]
*U6* snRNA		U6 snRNP component pre-mRNA splicing	[57,58]
*U3* snoRNA		Box C/D snoRNA pre-rRNA processing	[59]
*U8* snoRNA		Box C/D snoRNA pre-rRNA processing	[59]
*U14* snoRNA		Box C/D snoRNA pre-rRNA processing	[59]
*TERC RNA*	TR	Telomerase complex	[53]
**Gemini of Cajal Body**			
Proteins			
SMN1	GEMIN1	SMN complex Spliceosomal snRNP biogenesis	[60]
GEMIN2	SIP1	SMN complex Spliceosomal snRNP biogenesis	[61]
GEMIN3		SMN complex Spliceosomal snRNP biogenesis	[62]
GEMIN4		SMN complex Spliceosomal snRNP biogenesis	[63]
GEMIN5		SMN complex Spliceosomal snRNP biogenesis	[64]
GEMIN6	SIP2	SMN complex Spliceosomal snRNP biogenesis	[65]
GEMIN7	SIP3	SMN complex Spliceosomal snRNP biogenesis	[66]
GEMIN8		SMN complex Spliceosomal snRNP biogenesis	[67]
ZPR1		SMN complex SMN localization	[68]
**Histone Locus Body**			
Proteins			
NPAT	p220	Histone gene transcription	[69]
SLBP	HBP	Histone pre-mRNA 3' end processing	[70]
LSm10		U7 snRNP component Histone pre-mRNA 3' end processing	[70]
LSm11		U7 snRNP component Histone pre-mRNA 3' end processing	[70,71]
FLASH	CASP8AP2	Histone gene transcription	[72]
NELF A	WHSC2	NELF complex Histone pre-mRNA 3' end processing	[73]
NELF B	COBRA1	NELF complex Histone pre-mRNA 3' end processing	[73]
NELF C/D	TH1L	NELF complex Histone pre-mRNA 3' end processing	[73]
NELF E	RDBP	NELF complex Histone pre-mRNA 3' end processing	[73]
HiNF-P		Transcription	[74]
ZPR1		NPAT, SMN, coilin localization to HLBs	[75]
Coilin		Function unknown	[76]
RNA			
*U7* snRNA		U7 snRNP component Histone pre-mRNA 3' end processing	[71]
DNA			
Histone loci		Replication-dependent histone genes	[71,77]
**Paraspeckle**			
Proteins			
NONO	p54nrb	DBHS family	[78]
PSP1	PSPC1	DBHS family	[78]
PSP2	CoAA RBM14	Transcription Splicing	[78]
SFPQ	PSF	DBHS family	[79]
CFIm68	CPSF6	CFI_m_ complex pre-mRNA 3' end processing	[80]
RNAs			
*NEAT1* RNA	*MEN ε/β* *VINC*	Mammal-specific long ncRNA	[81,82,83]
*Ctn* RNA		Mouse-specific isoform transcribed from the *Slc7a2* locus	[79]

Abbreviations: CFI_m_, mammalian cleavage factor I; CPSF, cleavage and polyadenylation specificity factor; CSTF, cleavage stimulation factor; DBHS, *Drosophila* behavior/human splicing; HLB, histone locus body; mRNA, messenger RNA; ncRNA, non-coding RNA; NELF, negative elongation factor; NMD, nonsense-mediated mRNA decay; NPAT, nuclear protein ataxia telangiectasia locus; pre-mRNA, precursor messenger RNA; pre-rRNA, precursor ribosomal RNA; scaRNA, small Cajal body-specific RNA; S/MAR, scaffold/matrix attachment region; SMN, survival of motor neuron; snoRNA, small nucleolar RNA; snoRNP, small nucleolar ribonucleoprotein; snRNA, small nuclear RNA; snRNP, small nuclear ribonucleoprotein; SRSF, serine/arginine-rich splicing factor.

**Table 2:** Selected examples of model systems deficient for nuclear body components.

Nuclear Body Component	Model System	Experimental Manipulation	Phenotype	References
**Nucleolus**				
Proteins				
?	African clawed frog	Anucleolate mutant	Absence of rRNA synthesis Developmental arrest	[8]
Nucleophosmin	HeLa cells	Transduction of NPM shuttling defective mutants	⬇ Nuclear export of 40S and 60S ribosomal subunits ⬇ Protein synthesis	[14]
Nucleolin	HeLa cells	siRNA KD of *NCL*	⬇ RNA Pol I transcription	[16]
Fibrillarin	Budding yeast	Conditionally lethal allele of *NOP1*	⬇ 18S rRNA ⬇ Ribosome biogenesis ⬇ Growth rate	[84]
Dyskerin	HeLa cells	siRNA KD of *DKC1*	⬇ Pseudouridylation of rRNAs ⬇ Ribosome affinity for substrate ⬇ Translational fidelity	[85]
Dyskerin	Cultured mouse embryonic fibroblasts	Hypomorphic allele of *Dkc1*	⬇ Pseudouridylation of rRNAs ⬇ Ribosome affinity for substrate ⬇ Translational fidelity	[85]
Dyskerin	Budding yeast	Hypomorphic allele of *CBF5*	⬇ Pseudouridylation of rRNAs ⬇ Ribosome affinity for substrate ⬇ Translational fidelity	[85]
RNA Pol I	Human peripheral blood lymphoblasts and cultured fibroblasts	Actinomycin Dtreatment	Condensation of nucleolar chromatin Coilin enriched in nucleolar caps	[86]
RPS19	DBA patient-derived CD34^-^ cells from bone marrow	None	⬇Mature 40S ribosomal subunit pre-rRNA processing defect	[87]
RPS19	Human erythroleukemia cells	siRNA KD of *RPS19*	pre-rRNA processing defect	[87]
RNA				
*U3* snoRNA	Budding yeast	Conditionally repressible *SNR17A* gene	⬇ 18S rRNA ⬇ Growth rate	[88]
**Nuclear Speckle**				
Proteins				
SRSF1	HeLa cells	siRNA KD of *SRSF1*	⬇ Nuclear speckle localization of pre-mRNA processing factors Enlarged nuclear speckles	[89]
SRSF1	Human osteosarcoma cells	siRNA KD of *SRSF1*	⬇ Transcription	[89]
SRSF2	Human osteosarcoma cells	siRNA KD of *SRSF2*	⬇ Transcription	[89]
CLK	Human epidermoid carcinoma cells	Overexpression of murine CLK	⬇ pre-mRNA splicing Disrupted nuclear speckles	[90]
CLK	Human epidermoid carcinoma cells	Overexpression of catalytically inactive murine CLK	Retention of hypophosphorylated SRSF proteins in nuclear speckles	[90]
PRPF6	HeLa cells	siRNA KD of *PRPF6*	⬇ *MALAT1* RNA ⬇ Localization of *MALAT1* to nuclear speckles Nuclear speckles not disrupted	[91]
SON	HeLa cells	siRNA KD of *SON*	⬇ Localization of *MALAT1* to nuclear speckles	[91]
SON	HeLa cells	siRNA KD of *SON*	⬇ Localization of pre-mRNA processing factors to nuclear speckles ⬇ Localization of core EJC proteins to nuclear specklesCell cycle arrest	[92]
Aly/REF	HeLa cells	siRNA KD of *ALYREF*	⬇ mRNA export	[93]
Aly/REF	HeLa cells	siRNA KD of *ALYREF*	⬇ mRNA export ⬇ Poly(A)+ RNA in nuclear speckles	[94]
RNAs				
*MALAT1*	HeLa cells	ASO KD of *MALAT1*	⬇ Nuclear speckle integrityAberrant alternative splicing ⬇ SRSF1 and SRSF2 Altered ratio of dephosphorylated to phosphorylated SRSF proteins	[91]
*Malat1*	Mouse	KO	Localization of nuclear speckle proteins unaffected Viable and fertile	[95,96]
*U1* snRNA	HeLa cells	ASO KD of *U1* snRNA	Splicing inhibition Enlarged speckles ⬇ Number of nuclear speckles ⬇ Transcription	[97]
*U2* snRNA	HeLa cells	ASO KD of *U2* snRNA	Splicing inhibition Enlarged speckles ⬇ Number of nuclear speckles ⬇ Transcription	[97]
**Nuclear Stress Body**				
Proteins				
HSF1	Cultured human embryonic kidney cells	shRNA KD of *HSF1*	⬇ Satellite III transcription	[98]
HSF1	HeLa cells	shRNA KD of *HSF1*	⬇ HSF2 protein No nSBs upon heat shock	[98]
HSF2	Cultured human embryonic kidney cells	siRNA KD of *HSF2*	⬇ Satellite III transcription No effect on HSF1 localization to nSBs upon heat shock	[98]
**Transcription Factory**				
Proteins				
RNA Pol I	Human cells	Actinomycin D treatment	Coilin enriched in nucleolar caps	[86]
RNA Pol II	Human cells	α-Amanitin or DRB treatment	Chromatin decondensation Loss of nucleolar structure	[86]
RNA Pol II	HeLa cells	α-Amanitin treatment	⬇ BrUTP incorporation	[99,100]
RNA Pol II	Canine kidney cells	α-Amanitin, DRB, H8, or actinomycin D treatment	Redistribution of RNA Pol II to enlarged nuclear speckles	[36]
RNA Pol III	HeLa cells	α-Amanitin treatment	#x2B07; BrUTP incorporation	[100]
**Cajal Body**				
Proteins				
Coilin	Mouse	KO	⬇ Viability Residual CBs present No SMN localization to CBs No Sm protein localization to CBs	[101,102]
Coilin	African clawed frog oocyte	Coilin removal by IP	CBs present No Sm protein localization to CBs	[103]
Coilin	Fruit fly	Amorphic alleles of *coil*	No CBs No effect on HLBs Viable and fertile	[76]
Coilin	Fruit fly	Amorphic alleles of *coil*	No CBs No scaRNA localization to CBs snRNAs properly modified	[104]
Dyskerin	DKC patient-derived iPSCs	None	⬇ Telomerase assembly ⬇ Telomere synthesis	[105]
TERT	DKC patient-derived iPSCs	None	⬇ Telomerase levels ⬇ Telomere synthesis	[105]
TCAB1	DKC patient-derived iPSCs	None	Telomerase activity unaffected ⬇ Telomerase localization to CBs ⬇ Telomere synthesis	[105]
TCAB1	HeLa cells	shRNA KD of *WRAP53*	⬇ TERC localization to CBs ⬇ TERC localization to telomeres ⬇ Telomere synthesis	[54]
NOPP140	Cultured SMA patient-derived fibroblasts	None	⬇ Localization of dyskerin and GAR1 to CBs ⬇ Localization of NOPP140 correlated with disease severity	[106]
SMN1	Cultured SMA patient-derived lymphoblasts and fibroblasts	None	⬇ Gems ⬇ SMN protein	[107,108]
SMN1	HeLa cells	siRNA KD of *SMN1*	Defects in CB formation snRNPs absent in coilin foci	[109]
SMN1	HeLa cells	siRNA KD of *SMN1*	⬇ Gem-associated proteins ⬇ snRNP assembly Gems absent	[110,111]
SMN1	Mouse	Neuron-specific exon 7 deletion of *Smn1*	Coilin aggregates Gems absent SMA phenotype	[112]
SMN1	Mouse	SMA models varying in disease severity	⬇ SMN complex proteins ⬇ snRNP biogenesis Correlation with disease severity	[113]
SMN1	Mouse	Human SMN2 and SMN∆7 expression in *Smn1^-/-^* background	⬇ SMN complex proteins ⬇ snRNP biogenesis Tissue-specific snRNA alterations Widespread splicing defects SMA phenotype	[114]
SMN1	Fission yeast	Temperature-sensitive degron allele of *smn1*	Differential snRNP biogenesis Splicing defects	[115]
**Gemini of Cajal Body**				
Proteins				
SMN1	Cultured SMA patient-derived lymphoblasts and fibroblasts	None	⬇ Gems ⬇ SMN protein	[107,108]
SMN1	HeLa cells	siRNA KD of *SMN1*	Defects in CB formation snRNPs absent in coilin foci	[109]
SMN1	HeLa cells	siRNA KD of *SMN1*	⬇ Gem-associated proteins ⬇ snRNP assembly Gems absent	[110,111]
SMN1	Mouse	Neuron-specific exon 7 deletion of *Smn1*	Coilin aggregates Gems absent SMA phenotype	[112]
SMN1	Mouse	SMA models varying in disease severity	⬇ SMN complex proteins ⬇ snRNP biogenesis Correlation with disease severity	[113]
SMN1	Mouse	Human SMN2 and SMN∆7 expression in *Smn1^-/-^* background	⬇ SMN complex proteins ⬇ snRNP biogenesis Tissue-specific snRNA alterations Widespread splicing defects SMA phenotype	[114]
SMN1	Fission yeast	Temperature-sensitive degron allele of *smn1*	Differential snRNP biogenesis Splicing defects	[115]
GEMIN2	HeLa cells	siRNA KD of *GEMIN2*	⬇ GEMIN3 protein ⬇ snRNP biogenesis	[110,111]
GEMIN3	HeLa cells	siRNA KD of *GEMIN3*	⬇ GEMIN4 protein ⬇ snRNP biogenesis	[110,111]
GEMIN3	Fruit fly	LOF alleles of *Gem3*	⬇ SMN protein Motor defects Larval lethal	[116]
GEMIN4	HeLa cells	siRNA KD of *GEMIN4*	⬇ GEMIN3 protein ⬇ snRNP biogenesis	[110,111]
GEMIN5	HeLa cells	siRNA KD of *GEMIN5*	No effect on gems No effect on snRNP biogenesis	[110,111]
GEMIN6	HeLa cells	siRNA KD of *GEMIN6*	No effect on gems ⬇ snRNP biogenesis	[110,111]
GEMIN7	HeLa cells	siRNA KD of *GEMIN7*	⬇ snRNP biogenesis	[111]
ZPR1	HeLa cells	ASO KD of *ZPR1*	⬇ SMN localization to gems and CBs	[68]
ZPR1	Mouse	KO	⬇ Localization of SMN and coilin to gems and CBs Mislocalization of snRNPs Embryonic lethal	[117]
ZPR1	Mouse	Heterozygous KO	Mislocalization of SMN Motor neuron degeneration Motor defects	[118]
ZPR1	Cultured mouse motor neuron-like cells	siRNA KD of *Zpr1*	⬇ Localization of SMN to gems and CBs Mislocalization of snRNPs Axonal defects	[117]
**Histone Locus Body**				
Proteins				
NPAT	Mouse	Retroviral insertion of *Npat*	Embryonic lethal	[119]
SLBP	Human osteosarcoma cells	siRNA KD of *SLBP*	Aberrant histone RNA processing S phase block ⬇ Cell proliferation ⬇ Histone mRNA and protein	[120]
SLBP	HeLa cells	shRNA KD of *SLBP*	Aberrant histone RNA processing	[73]
SLBP	Fruit fly	LOF alleles of *Slbp*	Aberrant histone pre-mRNA processing Female sterile Lethal	[121]
SLBP	Fruit fly	LOF allele of *Slbp*	No effect on HLBs	[122]
LSm10	Fruit fly	LOF alleles of *Lsm10*	Aberrant histone pre-mRNA processing No U7 snRNA localization to HLBs Lethal	[123]
LSm11	Fruit fly	LOF alleles of *Lsm11*	⬇ Lsm10 protein Aberrant histone pre-mRNA processing No *U7* snRNA localization to HLBs Lethal	[123]
FLASH	HeLa cells	shRNA KD of *CASP8AP2*	⬇ Histone mRNA ⬇ Histone protein S phase block	[124]
FLASH	Human breast cancer cells	shRNA KD of *CASP8AP2*	No HLBs	[124]
FLASH	Mouse	KO	Embryonic lethal	[125]
NELF E	HeLa cells	shRNA KD of *RDBP*	⬇ NELF complex proteins ⬇ Cell proliferation Aberrant histone RNA processing	[73]
HiNF-P	Human glioblastoma cells	ASO and siRNA KD of *HINFP*	⬇ Histone 4 gene expression ⬇ RNA Pol II and NPAT histone 4 promoter occupancy	[74]
HiNF-P	Mouse	Amorphic allele of *Hinfp*	⬇ Histone 4 gene expression Embryonic lethal	[126]
ZPR1	HeLa cells	siRNA and ASO KD of *ZPR1*	Mislocalization of SMN, NPAT, coilin and Sm proteins S phase block ⬇ Transcription	[75]
ZPR1	Mouse	KO	Embryonic lethal	[117]
Coilin	Human breast cancer cells	shRNA KD of *COIL*	No effect on HLBs	[124]
Coilin	Cultured mouse embryonic fibroblasts	shRNA KD of *Coil*	No effect on HLBs	[124]
Coilin	Fruit fly	Amorphic alleles of *coil*	No CBs No effect on HLBs Viable and fertile	[76]
RNA				
*U7* RNA	Fruit fly	Null alleles of *U7* snRNA	Aberrant histone pre-mRNA processing No effect on HLBs No Lsm10 and Lsm11 localization to HLBs Viable, but sterile	[122,123,127]
DNA				
Histone locus	Fruit fly	Histone locus deletion mutant *Df(2L)DS6*	Smaller proto-HLBs	[122,128]
**Paraspeckle**				
Proteins				
NONO	HeLa cells	siRNA KD of *NONO*	⬇ Paraspeckles	[83]
NONO	Mouse	Chondrocyte lineage-specific expression of truncated murine NONO	Dwarfism ⬇ Chondrogenesis	[129]
NONO	Cultured mouse chondrogenic cells	siRNA KD of *Nono*	⬇ Sox9-dependent *Col2a1* promoter activity and expression	[129]
NONO	Cultured mouse embryonic fibroblasts	siRNA KD of *Nono*	⬇ Circadian rhythm	[130]
NONO	Fruit fly	Hypomorphic allele of *nonA*	⬇ Circadian rhythm	[130]
PSP1	HeLa cells	siRNA KD of *PSPC1*	No effect on paraspeckles	[83]
SFPQ	HeLa cells	siRNA KD of *SFPQ*	No paraspeckles	[83]
**Paraspeckle**				
RNAs				
*NEAT1*RNA	HeLa cells	siRNA or ASO KD of *NEAT1*	No paraspeckles	[81,82,83,131]
*NEAT1* RNA	Human osteosarcoma cells	shRNA KD of *NEAT1*	No paraspeckles Normal circadian rhythm	[132]
*Neat1* RNA	Mouse	KO	No paraspeckles Viable and fertile	[133]
*Ctn* RNA	Cultured mouse mammary tumor and macrophage cells	ASO KD of *Ctn*	No effect on paraspeckles ⬇ *Ctn* RNA and *Cat2* mRNA	[79]

Abbreviations: ASO, antisense oligonucleotide; BrUTP, 5-bromouridine 5'-triphosphate; CB, Cajal body; DBA, Diamond-Blackfan anemia; DKC, dyskeratosis congenita; DRB; 5,6-dichloro-1-β-D-ribofuranosylbenzimidazole; EJC, exon junction complex; gem, Gemini of Cajal body; HLB, histone locus body; HSF, heat shock factor; IP, immunoprecipitation; iPSC, induced pluripotent stem cell; KD, knockdown; KO, knockout; LOF, loss of function; mRNA, messenger RNA; NELF, negative elongation factor; NPAT, nuclear protein ataxia telangiectasia locus; nSB, nuclear stress body; pre-mRNA, precursor messenger RNA; pre-rRNA, precursor ribosomal RNA; RNA Pol, RNA polymerase; rRNA, ribosomal RNA; scaRNA, small Cajal body-specific RNA; shRNA, short hairpin RNA; siRNA, small interfering RNA; SMA, spinal muscular atrophy; SMN, survival of motor neuron; snRNA, small nuclear RNA; snRNP, small nuclear ribonucleoprotein; SRSF, serine/arginine-rich splicing factor.

#### 2.1.4. Mechanisms of Gene Expression and the Nucleolus

The nucleolus regulates gene expression by modulating protein production via ribosome biogenesis. Ribosome biogenesis is critical to cellular function, growth and response to stimuli. The multifunctional phosphoproteins, nucleophosmin and nucleolin, regulate ribosome biogenesis at several levels. Nucleophosmin specifically interacts with ribosomal DNA (rDNA) and regulates the transcription of rDNA as a histone chaperone [134]. Furthermore, it functions as the rate-limiting nuclear export chaperone for the precursor 40S (pre-40S) and precursor 60S (pre-60S) ribosomal subunits [14]. Nucleolin interacts with rDNA, maintains the open conformation of the rDNA genes, regulates the transcription of the rDNA genes and functions in rRNA processing [15,135]. The snoRNP complexes post-transcriptionally modify rRNA; these modifications affect the secondary structure that influences the stability, interactions and catalytic functionality of the rRNA. At least one study has demonstrated that a defect in the pseudouridylation of rRNAs leads to decreased ribosomal ligand binding and translational fidelity [85]. The biogenesis of ribosomes, therefore, requires the function of several proteins to transcribe rDNA, process pre-rRNA, assemble rRNA with ribosomal proteins and export the pre-40S and pre-60S ribosomal subunits.

In addition to the modulation of gene expression through ribosome biogenesis, the nucleolus modulates protein post-translational modifications, such as ubiquitination and SUMOylation. Ubiquitination of transcription termination factor 1 (TTF1) and p53 by ubiquitin ligase MDM2 regulates ribosome biogenesis and p53 regulation, respectively [136,137], and deubiquitination of the largest subunit of RNA Pol I by conserved yeast deubiquitinating enzyme, Ubp10, mediates RNA Pol I stability [138]. SUMOylation of NOP58 is critical for box C/D snoRNA binding and localization of newly transcribed snoRNAs to the nucleolus [139], and deSUMOylation of nucleophosmin by sentrin-specific protease 3 (SENP3) is needed for pre-rRNA processing [140]. 

Sequestration of proteins is another mechanism by which the nucleolus modulates expression of gene products. Proteins may be stabilized through sequestration of degradation factors to the nucleolus. For example, sequestration of ubiquitin ligases MDM2, and von Hippel-Lindau disease tumor suppressor (VHL) enhances the stability of their targets p53 and hypoxia-inducible factor 1 alpha (HIF1alpha), respectively [141,142]. Deactivation of a complex through the physical separation of its subunits has also been observed; tumor suppressor, p14ARF, inhibits the transcriptional activity of HIF1 through the sequestration of its alpha subunit to the nucleolus [143]. Finally, protein activity can be modulated by sequestration from the local environment in which it is active. For instance, the transcription repressor activity of DAXX is inhibited upon sequestration of DAXX to the nucleolus [144]. 

#### 2.1.5. Human Diseases Associated with the Nucleolus

Several human disorders associate with mutations in genes encoding nucleolar proteins (Table 3). Mutations in genes encoding the two RNA Pol I subunits, POLR1C and POLR1D, and in the nucleolar protein Treacher Collins-Franceschetti syndrome 1 (TCOF1, also known as treacle) cause the craniofacial disorder, Treacher Collins syndrome (OMIM 248390, OMIM 613717 and OMIM 154500). Additionally, dysregulation of RNA Pol I transcription in nucleoli is frequently observed in cancer [145]. Mutations in genes encoding for the 40S and 60S ribosomal subunits lead to Diamond-Blackfan anemia (OMIM 105650, OMIM 610629, OMIM 612527, OMIM 612528, OMIM 612561, OMIM 612562, OMIM 612563, OMIM 613308, OMIM 613309 and OMIM 614900). Finally, mutations in *WRN* and *BLM*, which encode nucleolar DNA helicases, cause Werner syndrome (OMIM 277700) and Bloom syndrome (OMIM 210900), respectively. 

**Table 3:** Selected human diseases associated with mutations in genes encoding nuclear body components.

Gene	Human Disease	OMIM No.	Phenotype	Disease Gene Identification References
**Nucleolus**				
* TCOF1*	Treacher Collins syndrome 1	154500	Craniofacial abnormalities	[146]
* POLR1D*	Treacher Collins syndrome 2	613717	Craniofacial abnormalities	[147]
* POLR1C*	Treacher Collins syndrome 3	248390	Craniofacial abnormalities	[147]
* RPS19*	Diamond-Blackfan anemia 1	105650	Hypoplastic anemia	[148]
* RPS24*	Diamond-Blackfan anemia 3	610629	Hypoplastic anemia	[149]
* RPS17*	Diamond-Blackfan anemia 4	612527	Hypoplastic anemia	[150]
* RPL35A*	Diamond-Blackfan anemia 5	612528	Hypoplastic anemia	[151]
* RPL5*	Diamond-Blackfan anemia 6	612561	Hypoplastic anemia	[152]
* RPL11*	Diamond-Blackfan anemia 7	612562	Hypoplastic anemia	[152]
* RPS7*	Diamond-Blackfan anemia 8	612563	Hypoplastic anemia	[152]
*RPS10*	Diamond-Blackfan anemia 9	613308	Hypoplastic anemia	[153]
* RPS26*	Diamond-Blackfan anemia 10	613309	Hypoplastic anemia	[153]
* RPL26*	Diamond-Blackfan anemia 11	614900	Hypoplastic anemia	[154]
* WRN*	Werner syndrome	277700	Premature aging syndrome	[155]
* BLM*	Bloom syndrome	210900	Growth deficiency Cancer predisposition	[156]
**Nuclear Speckle**				
*PRPF3*	Retinitis pigmentosa 18	601414	Retinal degeneration	[157]
*PRPF6*	Retinitis pigmentosa 60	613983	Retinal degeneration ⬇ pre-mRNA splicing	[158]
*PRPF8*	Retinitis pigmentosa 13	600059	Retinal degeneration	[159]
* SNRNP200*	Retinitis pigmentosa 33	610359	Retinal degeneration ⬇ *U4*/*U6* snRNA unwinding	[160]
*EFTUD2*	Mandibulofacial dysostosis, Guion-Almeida type	610536	Facial dysmorphism Progressive microcephaly Developmental delay Speech delay	[161]
*RBM8A*	Thrombocytopenia-absent radius syndrome	274000	Platelet reduction Radial bone aplasia	[162]
**Transcription Factory**				
* POLR1D*	Treacher Collins syndrome 2	613717	Craniofacial abnormalities	[147]
* POLR1C*	Treacher Collins syndrome 3	248390	Craniofacial abnormalities	[147]
*POLR3A*	Hypomyelinating leukodystrophy 7	607694	Hypomyelination Motor dysfunction ± Abnormal dentition ± Hypogonadism	[163,164]
*POLR3B*	Hypomyelinating leukodystrophy 8	614381	Hypomyelination Motor dysfunction ± Abnormal dentition ± Hypogonadism	[164,165]
*MED12*	Lujan-Fryns syndrome	309520	Marfanoid habitus Intellectual disability	[166]
*MED12*	Opitz-Kaveggia syndrome	305450	Facial dysmorphism Hypotonia Intellectual disability	[167]
*MED17*	Postnatal progressive microcephaly with seizures and brain atrophy	613668	Progressive microcephaly Brain atrophy Developmental retardation	[168]
*MED23*	Mental retardation, autosomal recessive 18	614249	Intellectual disability	[169]
*MED25*	Charcot-Marie-Tooth disease, type 2B2	605589	Distal muscle weakness and atrophy Sensory loss	[170]
*ERCC2*	Xeroderma pigmentosum, group D	278730	Sun sensitivity Increased risk for cancer	[171]
*ERCC2*	Cerebrooculofacioskeletal syndrome 2	610756	Microcephaly Ocular abnormalities Multiple joint contractures Dysmorphic features	[172]
*ERCC2*	Trichothiodystrophy	601675	Brittle hair and nails Ichthyotic skin Growth delay Intellectual disability	[173]
*ERCC3*	Xeroderma pigmentosum, group B	610651	Sun sensitivity Increased risk for cancer	[174]
*ERCC3*	Trichothiodystrophy	601675	Brittle hair and nails Ichthyotic skin Growth delay Intellectual disability	[175]
*GTF2H5*	Trichothiodystrophy	601675	Brittle hair and nails Ichthyotic skin Growth delay Intellectual disability	[176]
**Cajal Body**				
*SMN1*	Spinal muscular atrophy, type I Spinal muscular atrophy, type II Spinal muscular atrophy, type III Spinal muscular atrophy, type IV	253300 253550 253400 271150	Lower motor neuron degeneration ⬇ SMN protein ⬇ Gems	[177]
* DKC1*	Dyskeratosis congenita, X-linked	305000	Nail dystrophy Lacy skin pigmentation Oral leukoplakia ⬇ *TERC* RNA ⬇ Telomerase assembly ⬇ Telomerase activity	[178]
* DKC1*	Hoyeraal-Hreidarsson syndrome	300240	Growth delay Immunodeficiency Cerebellar hypoplasia	[179]
*NHP2*	Dyskeratosis congenita, autosomal recessive 2	613987	Nail dystrophy Lacy skin pigmentation Oral leukoplakia ⬇ Telomerase assembly	[180]
*NOP10*	Dyskeratosis congenita, autosomal recessive 1	224230	Nail dystrophy Lacy skin pigmentation Oral leukoplakia ⬇ Telomerase assembly	[181]
* TERT*	Dyskeratosis congenita, autosomal dominant 2 Dyskeratosis congenita, autosomal recessive 4	613989	Nail dystrophy Lacy skin pigmentation Oral leukoplakia ⬇ Telomerase activity	[182,183]
*WRAP53*	Dyskeratosis congenita, autosomal recessive 3	613988	Nail dystrophy Lacy skin pigmentation Oral leukoplakia ⬇ Telomerase localization to CBs	[184]
* TERC*	Dyskeratosis congenita, autosomal dominant 1	127550	Nail dystrophy Lacy skin pigmentation Oral leukoplakia	[185]
**Gemini of Cajal Body**				
*SMN1*	Spinal muscular atrophy, type I Spinal muscular atrophy, type II Spinal muscular atrophy, type III Spinal muscular atrophy, type IV	253300 253550 253400 271150	Lower motor neuron degeneration ⬇ SMN protein ⬇ Gems	[177]

Abbreviations: CB, Cajal body; gem, Gemini of Cajal body; OMIM, Online Mendelian Inheritance in Man; pre-mRNA, precursor messenger RNA; SMN, survival of motor neuron; snRNA, small nuclear RNA.

### 2.2. Nuclear Speckle

#### 2.2.1. Discovery

First described as *grumos hialinas* or transparent lumps by Santiago Ramón y Cajal in 1910 [186] and subsequently detected by electron microscopy and immunofluorescence [187,188], nuclear speckles or interchromatin granule clusters have a speckled distribution in the interchromatin regions of the nucleus. The similar distribution of spliceosomal snRNPs was a clue to the function of nuclear speckles in pre-mRNA splicing [189,190,191] and integration of transcription with pre-mRNA splicing [192]. 

#### 2.2.2. Key Components

Most constituents in the nuclear speckle are components of the spliceosome and function in pre-mRNA splicing; these include the spliceosomal snRNAs and several associated protein factors that comprise the snRNPs [33,34] (Table 1). Other constituents include heteronuclear RNPs (hnRNPs), cleavage and polyadenylation factors, protein kinases, such as CDC-like kinase (CLK), members of the exon junction complex (EJC) and structural proteins [33,34]. Two abundant RNAs in nuclear speckles are the long non-coding RNA (ncRNA) metastasis-associated lung adenocarcinoma transcript 1 (*MALAT1*) and polyadenylated (poly(A)+) RNA [38,39].

#### 2.2.3. Functions of Key Components

Members of the serine (S)/arginine (R)-rich splicing factor (SRSF) family, which are the predominant splicing factors found in the nuclear speckle [32], participate in constitutive and alternative splicing, as well as in transcription [193], nonsense-mediated mRNA decay (NMD), mRNA translation and genome stability [194]. They may also contribute to the integrity of the nuclear speckle, since RNA interference (RNAi)-mediated knockdown of *SRSF1* or *SRSF2* decreases localization of other pre-mRNA processing factors to nuclear speckles, enlarges nuclear speckles and decreases the transcription of some genes [89] (Table 2). Members of the spliceosomal snRNPs are involved in pre-mRNA splicing, while members of the cleavage and polyadenylation specificity factor (CPSF) complex facilitate pre-mRNA 3' end processing (Table 1).

Proteins, mago nashi homologue (MAGOH), eukaryotic initiation factor 4A III (eIF4AIII), RNA-binding protein S1 (RNPS1), Y14 and Aly/REF, which are members of the EJC, function in RNA surveillance, NMD and, in conjunction with nuclear speckle protein, RAE1, the nuclear export of mRNA [195] (Table 1). RNAi-mediated knockdown of Aly/REF decreases nuclear mRNA export and causes increased poly(A)+ RNA accumulation in nuclear speckles [94] (Table 2).

The long ncRNA *MALAT1*, which is retained in the nucleus, binds and regulates SRSF proteins and, thereby, modulates alternative splicing [91]. Despite this, however, *Malat1*-null mice are viable and fertile, and the cells and tissues tested from these animals have appropriately localized nuclear speckle proteins [95]. In contrast, knockdown of *MALAT1* in HeLa cells causes aberrant alternative splicing, decreased localization of pre-mRNA processing factors to nuclear speckles and a distorted ratio of dephosphorylated to phosphorylated pools of SRSF proteins [91]. Further studies are required to delineate this apparent functional difference.

#### 2.2.4. Mechanisms of Gene Expression and the Nuclear Speckle

The nuclear speckle regulates gene expression by possibly regulating transcription directly and via post-transcriptional mechanisms. Observations supporting a direct effect on transcription include (1) the enrichment of the periphery of nuclear speckles for the elongating form of RNA Pol II [36,196], (2) the facilitation of transcriptional elongation by the nuclear speckle protein, SRSF2 [193], and (3) the concurrent splicing and transcription of 80% of pre-mRNA [192]. Observations supporting a post-transcriptional effect on gene expression include (1) the modulation of constitutive and alternative splicing [94,192] and (2) the modulation of nuclear export, mRNA surveillance and post-translational modification. Exemplifying the latter, the nuclear speckle kinase, CLK, phosphorylates SRSF proteins to alter their intranuclear distribution [35], pre-mRNA splicing efficiency [197] and recruitment to transcription sites [198]. Lastly, nuclear speckles likely integrate transcription with mRNA export, since the nuclear speckle proteins, Aly/REF and U2AF65-associated protein 56 kDa (UAP56), function in both transcription and mRNA export [199].

Nuclear speckles may also directly modulate gene expression through interaction with the RNA Pol II complex [194], even though transcription does not occur within nuclear speckles [200]. 

#### 2.2.5. Human Diseases Associated with the Nuclear Speckle

Disorders associated with mutations in genes encoding nuclear speckle components include retinitis pigmentosa (OMIM 600138, OMIM 600059, OMIM 601414, OMIM 610359 and OMIM 613983), mandibulofacial dysostosis with microcephaly (OMIM 610536) and thrombocytopenia-absent radius syndrome (OMIM 274000) (Table 3). Several of these mutations likely affect spliceosome activity and pre-mRNA splicing [158,160] (Table 3). 

### 2.3. Nuclear Stress Body

#### 2.3.1. Discovery

First identified as foci of heat shock factor 1 in heat-stressed cells [40,201,202], nuclear stress bodies transiently form in response to various cellular stresses, such as heat shock, ultraviolet light and chemical agents, such as heavy metals, the amino acid analog azetidine and proteasome inhibitors. Although nuclear stress bodies have only been detected in primate cells [203], analogous stress-inducible structures have been observed in the cells of *Drosophila melanogaster* and *Caenorhabditis elegans* [204,205] (Table 4).

#### 2.3.2. Key Components of the Nuclear Stress Body

The nuclear stress body consists of both protein and non-protein components that modulate gene expression via transcription and RNA splicing. The protein components include the heat shock transcription factors, heat shock factor 1 (HSF1) and heat shock factor 2 (HSF2), scaffold attachment factor B (SAF-B), Src-associated in mitosis 68 kDa protein (Sam68) and the SRSF family members, SRSF1, SRSF7 and SRSF9 [40,41,42,43,201,206] (Table 1). RNA Pol II is also present in nuclear stress bodies [44]. The one identified non-protein component is the long satellite III ncRNA [44] (Table 1).

#### 2.3.3. Functions of Key Components

The heat shock transcription factors, HSF1 and HSF2, participate in the cellular response to stress. Present as inactive monomers in the cytoplasm, these transcription factors trimerize and translocate to the nucleus upon cellular stress [40]. They bind to heat shock elements (HSEs) present in the promoters of target genes and activate gene expression [207]. In addition to the genes encoding the heat shock proteins (HSPs), satellite III ncRNA, which is transcribed from the pericentric heterochromatic 9p12 locus, is also targeted [44,208]. In human cells, knockdown of *HSF1* impeded heat induction of nuclear stress bodies, HSF2 binding to DNA and satellite III transcription, whereas knockdown of *HSF2* had no effect on HSF1 localization to nuclear stress bodies and increased satellite III transcription [98] (Table 2). Thus, HSF2 binding to DNA is HSF1-dependent, and both HSF1 and HSF2 are required for the regulated expression of target genes.

SAF-B is an hnRNP that is involved in the transcriptional regulation of the gene encoding the heat shock protein, Hsp27 [209], as well as repression of estrogen receptor alpha-mediated transcription [210]. 

**Table 4:** Conservation of nuclear bodies across species.

Nuclear Body	Human *Homo sapiens*	Mouse *Mus musculus*	African clawed frog *Xenopus laevis*	Zebrafish *Danio rerio*	Fruit fly *Drosophila melanogaster*	Nematode *Caenorhabditis elegans*	Budding yeast *Saccharomyces cerevisiae*	References
Nucleolus	+	+	+	+	+	+	+^1^	[211,212,213,214,215]
Nuclear speckle	+	+	+^2^	ND	+	ND	−	[216,217,218,219]
Nuclear stress body	+	−	ND	ND	−^3^	−^4^	ND	[203,204,205]
Transcription factory	+	+	ND	ND	ND	ND	ND	[99,220]
Cajal body	+	+	−^5^	+	+	ND	+^6^	[71,216,221,222,223,224]
Gemini of Cajal body	+	+	ND	ND	−^7^	ND	ND	[60,225,226]
Histone locus body	+	ND	+^8^	−^9^	+	ND	ND	[71,77,223,227,228]
Paraspeckle	+	+	ND	ND	ND	ND	ND	[78,79]

Determination of the presence or absence of a nuclear body is based on the detection of bona fide, conserved, endogenous nuclear body markers in cultured cells or tissue of the species of interest. Studies that require further experimental validation have been noted. Abbreviations: +, presence of the nuclear body in the species of interest; −, absence of the nuclear body in the species of interest; ND, not determined. ^1^ The morphological, biochemical and electron microscopic studies demonstrating that the dense crescent in yeast is equivalent to the nucleolus were first performed in *Saccharomyces carlsbergensis*; ^2^ also known as the B snurposome in *Xenopus laevis*; ^3^ although nuclear stress bodies have not been observed in *Drosophila melanogaster*, the induction of *hsrω* transcripts and the formation of omega speckles upon heat shock is similar to the satellite III transcripts and nuclear stress bodies in human cells, respectively; ^4^ nuclear stress granule-like structures have been observed in *Caenorhabditis elegans* upon expression of physiological levels of a fluorescently tagged HSF1 fusion protein; ^5^ *Xenopus laevis* have Cajal body-like pearls that contain coilin and scaRNAs; however, they do not contain splicing snRNAs and are specifically associated with RNA polymerase III loci, unlike Cajal bodies in other species; ^6^ also known as the nucleolar body in *Saccharomyces cerevisiae*; ^7^ Gemini of Cajal bodies have been observed in *Drosophila melanogaster* larvae upon constitutive overexpression of a fluorescently tagged Gemin3 fusion protein; ^8^ also known as the C snurposome in *Xenopus laevis*; ^9^ nuclear bodies enriched for histone locus body components, *U7* snRNA and LSm11, and deficient for coilin have been observed in *Danio rerio* embryos upon injection of messenger RNA encoding fluorescently tagged zebrafish coilin or LSm11 and *in vitro* transcribed fluorescently labeled mouse *U7* snRNA. However, it is unknown whether these nuclear bodies also co-localize with the histone gene locus.

SRSF1, SRSF7 and SRSF9 participate in both constitutive and alternative splicing of pre-mRNA [197,229]. Additionally, SRSF1 processes microRNA [230]. The SRSF proteins have overlapping and distinct functions, differential expression and differential sequestration to nuclear stress bodies [43,197,231,232]. The alternative splicing regulator, Sam68, also accumulates in nuclear stress bodies upon cellular stress and likely mediates alternative splicing of transcripts in the nuclear stress body [233]

The satellite III transcripts are ncRNAs specific to nuclear stress bodies [44,234]. After being transcribed from pericentric heterochromatin at the 9q12 locus upon cellular stress, they remain associated with this genetic locus and are integral to the nuclear stress body that forms at the 9q12 locus [44,234]. The satellite III transcripts are required for the localization of proteins, such as SRSF1 and SRSF9, to the nuclear stress body [234]. Furthermore, the overexpression of satellite III transcripts has been shown to initiate the formation of nuclear stress bodies [235].

#### 2.3.4. Mechanisms of Gene Expression and the Nuclear Stress Body

Although nuclear stress bodies sequester factors that regulate expression of genes involved in the stress response, the role of nuclear stress bodies themselves in gene expression has not been delineated. Besides the sequestration of transcription and RNA processing factors, three observations suggest a role in the modulation of gene expression. First, RNA Pol II and acetylated histones localize to nuclear stress bodies following stress [44]. Second, the nuclear stress protein, HSF1, controls genome-wide histone deacetylation upon heat stress [236]. Third, the splicing factors associated with nuclear stress bodies have been implicated in alternative splicing [233].

#### 2.3.5. Human Diseases Associated with the Nuclear Stress Body

No human diseases have been described for mutations in genes encoding for nuclear stress body proteins or RNAs. 

### 2.4. Transcription Factory

#### 2.4.1. Discovery

Transcription factories were first observed and analyzed through biochemical studies and fluorescence and electron microscopy of the RNA polymerases and their nascent transcripts in HeLa cells [45,99]. These studies revealed that the RNA polymerases, and their nascent transcripts, are not diffusely scattered throughout the nucleus, but rather, are concentrated in a few thousand sites where transcription and RNA processing occur [45,99]. Although studies have provided evidence for stationary RNA polymerases and the existence of transcription factories [237], the recognition of transcription factories as a canonical nuclear body remains controversial. However, given their relevance to the regulation of gene expression, we review transcription factories.

#### 2.4.2. Key Components of the Transcription Factory

The three RNA polymerases are the defining components for their respective transcription factories. Other associated proteins include general transcription factors, the mediator complex, gene-specific regulatory factors, chromatin remodeling proteins, helicases, nucleic acid-binding proteins, RNPs and structural proteins [46] (Table 1). Transcription factories are, however, more than the simple co-localization of proteins, since they remain intact upon nucleolytic removal of chromatin and detergent extraction and in the absence of transcription [99,238,239]. 

#### 2.4.3. Gene Regulatory Functions of Key Components

The RNA polymerases and the general transcription factors comprise the machinery that allow for the transcription of genes. Gene-specific regulatory factors, such as transcriptional activators and repressors, control the expression of specific genes through interaction with the gene of interest. Chromatin remodeling proteins, in turn, modify the DNA accessibility to those transcription factors.

#### 2.4.4. Mechanisms of Gene Expression and the Transcription Factory

Transcription factories regulate gene expression by mediating transcription, concentrating the RNA polymerases and factors required for efficient transcription [240], coupling transcription with RNA processing [241] and modulating genomic structure [86]. 

#### 2.4.5. Human Diseases Associated with the Transcription Factory

Several disorders have been described for mutations in genes encoding transcription factory components (Table 3). Treacher Collins syndrome is associated with mutations of *POLR1C* and *POLR1D* (OMIM 248390 and OMIM 613717), and hypomyelinating leukodystrophy is associated with mutations of *POLR3A* and *POLR3B* (OMIM 607694 and OMIM 614381). No disorders have been associated with mutations of genes encoding RNA Pol II subunits. Mutations in genes encoding the mediator complex and general transcription factors cause congenital malformations, intellectual disability and features of defective DNA repair. Mediator complex mutations have been associated with Lujan-Fryns (OMIM 309520) and Opitz-Kaveggia (OMIM 305450) syndromes, postnatal progressive microcephaly with seizures and brain atrophy (OMIM 613668), mental retardation autosomal recessive 18 (OMIM 614249) and Charcot-Marie-Tooth disease type 2B2 (OMIM 605589). Mutations of the general transcription factor II H complex cause xeroderma pigmentosum group D (OMIM 278730) and group B (OMIM 610651), cerebrooculofacioskeletal syndrome 2 (OMIM 610756) and trichothiodystrophy (OMIM 601675). 

### 2.5. Cajal Body

#### 2.5.1. Discovery

Cajal bodies, also known as coiled bodies, are named after their discoverer, Santiago Ramón y Cajal [221], and are one of the first non-nucleolar nuclear bodies observed. Since its first sighting in the vertebrate neuron, Cajal bodies have been identified in tissues of diverse organisms, including vertebrates, invertebrates and plants [242]. Subsequent to the discovery of the Cajal body marker, coilin, understanding of the Cajal body and its role in regulating gene expression has accelerated [47,48,242,243,244].

#### 2.5.2. Key Components

The Cajal body is a site for the modification of small nuclear RNAs (snRNAs) and snoRNAs, as well as for the assembly and trafficking of RNPs. In addition to coilin, the Cajal body is enriched in spliceosomal small nuclear RNPs (snRNPs), snoRNPs, the telomerase RNP and in the factors that assemble and mature RNPs, as well as the survival of the motor neurons (SMN) complex [50,57,242,245] (Table 1). The multiprotein Integrator complex, which processes the 3' end of snRNAs [246] and maintains Cajal body integrity [247], may also be a member of the Cajal body [247].

#### 2.5.3. Functions of Key Components

Coilin, an abundant Cajal body protein of unknown function, interacts with several Cajal body components [248,249,250] and likely contributes to telomerase RNA biogenesis and snRNA processing [249,251]. Coilin deficiency is detrimental to Cajal body formation, localization of Cajal body proteins and viability of some model organisms (Table 2). Mediators of spliceosomal snRNP biogenesis also reside in the Cajal body; these include the small Cajal body-specific RNPs (scaRNPs), which direct the 2'-O-methylation and pseudouridylation of the snRNAs [56], and the SMN complex, which facilitates the nuclear import and localization of snRNAs and Sm proteins, as well as their assembly into spliceosomal snRNPs [61] (Table 1). Maturation of spliceosomal snRNPs in the Cajal body occurs prior to deposition of snRNPs in the nuclear speckle [252,253,254]. Similar to the snRNPs, many snoRNPs also assemble and mature in the Cajal body prior to their transport to the nucleolus [50,255]. Members of the telomerase RNP complex found in the Cajal body include the proteins, dyskerin, GAR1, NHP2, NOP10, telomerase reverse transcriptase (TERT), telomerase Cajal body protein 1 (TCAB1), and the telomerase RNA component (*TERC*) RNA [51,52,53,54] (Table 1). Deficiency of these components leads to reduced assembly, activity and/or localization of the telomerase RNP in the Cajal body and also to decreased telomere synthesis and loss of self-renewal [54,105,184,256] (Table 2). 

#### 2.5.4. Mechanisms of Gene Expression and the Cajal Body

snRNP biogenesis is one mechanism by which the Cajal body regulates gene expression. The Cajal body mediates snRNP biogenesis at many levels, including snRNA post-transcriptional modification, snRNA transcription and snRNP assembly. The scaRNPs of the Cajal body mediate the post-transcriptional modifications of 2'-O-methylation and pseudouridylation of snRNA [56]. The functional significance of the 2'-O-methylation and pseudouridylation of snRNA and snoRNAs is unclear; however, they likely affect the secondary structure of the RNAs and, thereby, their stability, RNA interactions, protein interactions and catalytic function [257,258]. Implicating Cajal bodies in snRNA gene expression, Cajal bodies co-localize with snRNA genes and transcripts [259,260,261]. Suggesting that the Cajal body is also involved in the processing of the spliceosomal snRNAs, coilin has been shown to have RNase activity with the *U2* snRNA primary transcript *in vitro*, and knockdown of coilin abrogates this activity [251]. Finally, the Cajal body participates in the assembly of snRNPs [253,262] and, thereby, indirectly contributes to precursor messenger RNA (pre-mRNA) splicing.

The Cajal body also facilitates the biogenesis and localization of another RNP, the telomerase complex. The telomerase complex is required for telomere synthesis. Evidence suggesting a role for the Cajal body in telomere maintenance includes the Cajal body-dependent assembly and localization of the telomerase complex to the telomeres during the S phase [54,105,263,264,265,266], the failure of pluripotent stem cells derived from dyskeratosis congenita patients with *TCAB1* mutations to lengthen telomeres [105] and the potential processing of telomerase RNA by coilin [249].

#### 2.5.5. Human Diseases Associated with the Cajal Body

Although Cajal body dysfunction has not been definitively implicated in human disease, mutations of several Cajal body components have been (Table 3). Functional deficiency of SMN1 is the molecular cause of spinal muscular atrophy (SMA) (OMIM 253300, OMIM 253550, OMIM 253400 and OMIM 271150) [107,177], a degenerative disorder of spinal cord motor neurons [267,268]. Mutations in genes encoding for telomerase complex members lead to the premature aging disorder, dyskeratosis congenita (OMIM 305000, OMIM 613987, OMIM 224230, OMIM 613989, 613988 and OMIM 127550).

### 2.6. Gemini of Cajal Body

#### 2.6.1. Discovery

In a study of the subcellular localization of the SMN1 protein, Liu and colleagues observed a novel nuclear structure similar in size, number, response to metabolic conditions and cell cycle behavior to Cajal bodies [60]. However, because it lacked the coilin protein characteristic of Cajal bodies, it was named the Gemini of Cajal body (also known as gems) [60].

#### 2.6.2. Key Components of the Gemini of Cajal Body

The identified components of gems include the SMN complex and ZPR1 (Table 1). The SMN complex consists of the SMN1 protein (also known as GEMIN1) and the gem-associated proteins 2–8 (GEMIN2–8). 

#### 2.6.3. Functions of Key Components

The SMN complex is required for spliceosomal snRNP biogenesis [61], as well as for transcription [269,270] and translation [271]. The gem-associated proteins, GEMIN2–8, also found in the Cajal body, are integral to the SMN complex and interact with other members of the SMN complex and/or the Sm proteins. GEMIN5 defines the specificity of the complex through the recognition of snRNAs [272]. Based on RNAi-mediated knockdown studies, the other GEMIN proteins participate in spliceosomal snRNP assembly and recruitment of other SMN complex members [110,111] (Table 2).

ZPR1, a highly conserved protein [273,274], is critical for viability in fission and budding yeast and in mice [68,117,273]. It interacts with and is required for the localization of SMN [68,117,273]. The interaction between ZPR1 and SMN is disrupted in SMA patient tissues, and mice heterozygous for *Zpr1* have motor defects, progressive motor neuron degeneration and mislocalization of the SMN protein [68,118] (Table 2). 

#### 2.6.4. Mechanisms of Gene Expression and the Gemini of Cajal Body

The precise function of gems is unknown; however, gems may be the nuclear domain responsible for further maturation, storage or recycling of snRNPs. Gems associate with Cajal bodies, and translocation of SMN between the two is mediated, at least in part, by post-translational modifications of coilin [275]. Methylation of the coilin arginine (R)- and glycine (G)-rich (RG) box motif increases its affinity for SMN, thereby, localizing SMN complexes to Cajal bodies; conversely, hypomethylation of the coilin RG box motif decreases its affinity for SMN, releasing SMN complexes to gems [275]. This dynamic molecular switch may represent an alternative pathway for snRNP biogenesis in gems and have downstream effects on pre-mRNA splicing. Further studies delineating additional gem components, their functions and their relation to Cajal bodies are necessary to address these issues.

#### 2.6.5. Human Diseases Associated with the Gemini of Cajal Body

Aside from spinal muscular atrophy, which was discussed above in the section on Cajal bodies, no human diseases have been specifically attributed to gems. The reduction of both SMN protein and the number of gems correlates with the clinical severity of SMA patients [107,108] (Table 3). Within model organisms, SMN deficiency also reduces the numbers of gems and alters spliceosomal snRNP biogenesis, pre-mRNA splicing and tissue-specific snRNA composition [110,111,112,113,114,115,276] (Table 2). 

### 2.7. Histone Locus Body

#### 2.7.1. Discovery

Searching for a vertebrate Cajal body equivalent in *Drosophila melanogaster*, Liu and colleagues found a nuclear body containing canonical Cajal body components that consistently co-localized with the histone gene locus [71]. Subsequent studies also identified these in *Xenopus laevis* and human nuclei, suggesting a conserved distinct nuclear compartment (Table 4). They therefore named this compartment the histone locus body [77,227,228]. 

#### 2.7.2. Key Components of the Histone Locus Body

The histone locus body components facilitate replication-dependent histone gene expression (Table 1). Components dedicated to this function are the nuclear protein, ataxia-telangiectasia locus (NPAT) protein, the stem-loop binding protein (SLBP), the U7 spliceosomal snRNP-specific components, like Sm proteins, LSm10 and LSm11, and the U7 spliceosomal snRNA, as well as FLICE-associated huge protein (FLASH) [69,70,71]. Components not dedicated solely to replication-dependent histone gene expression include members of the negative elongation factor (NELF) complex, histone nuclear factor P (HiNF-P), zinc finger protein 1 (ZPR1) and coilin [70,73,74,75,76]. 

#### 2.7.3. Functions of Key Components

During the S phase of the cell cycle, the replication-dependent histone genes are transcribed into RNAs that have a 3' untranslated region (UTR) with a highly conserved RNA hairpin element in lieu of a poly(A) tail. The histone locus body components contribute to the transcription, RNA processing, export, translation and degradation of the replication-dependent histone genes by linking their expression to the cell cycle. Cyclin E/Cdk2, which regulates the G1/S transition, phosphorylates the histone body component, NPAT, following its localization within the histone locus body by a ZPR1-dependent process [69,75]. Phosphorylated NPAT, in turn, activates replication-dependent histone gene expression by recruiting transcriptional activators to the histone gene promoters [69,74,124]. In cultured cells, dysregulation of NPAT or ZPR1 decreases histone gene expression, alters histone locus body protein localization, decreases the fidelity of histone RNA processing and impedes cell cycle progression; deficiency of either protein causes embryonic lethality in mice [117,119] (Table 2). 

Cell cycle regulation of SLBP expression also contributes to the cell cycle-dependent expression of the replication-dependent histone genes; SLBP is increased in expression shortly prior to the S phase, highly expressed during the S phase and degraded at the end of the S phase [69,277]. Processing the 3' ends of histone pre-mRNAs within the histone locus body requires SLBP, the U7 snRNP, the NELF complex and the histone pre-mRNA cleavage complex (HCC), which is comprised of FLASH, LSm11, symplekin, cleavage stimulation factor 64 (CSTF64) and all subunits of the CPSF complex [277]. SLBP binds the conserved RNA hairpin element at the 3' UTR and stabilizes the interaction of histone pre-mRNA with the U7 snRNP; SLBP also participates in the nuclear export, translation and degradation of histone mRNA [120,278,279]. After binding the histone downstream element (HDE) in the 3' UTR, the U7 snRNP and SLBP recruit the HCC, and the CPSF73 endonuclease cleaves the 3' end of the histone pre-mRNA. Deficiency of SLBP, the U7 snRNP or the NELF complex causes aberrant histone RNA 3' end processing, but does not affect the formation of histone locus bodies (Table 2).

Coilin, the classical marker of Cajal bodies, is also found in histone locus bodies [76]. Its function within histone locus bodies is currently undefined. RNAi-mediated knockdown and null alleles of coilin orthologues in mammalian cells and *Drosophila,* respectively, have no effect on the formation and function of histone locus bodies [76,124].

The invariant co-localization of histone locus bodies with the histone locus is a defining feature of this nuclear body. The histone locus itself, therefore, may be required for its structural integrity, and in fact, smaller histone locus bodies have been observed in *Drosophila* embryos deficient for the histone locus [122] (Table 2).

#### 2.7.4. Mechanisms of Gene Expression and the Histone Locus Body

The histone locus body regulates expression of replication-dependent histone genes by concentrating the required protein complexes and RNA components at the histone gene locus during the appropriate phase of the cell cycle. This is accomplished, at least in part, through the cell cycle regulation of NPAT and SLBP [277]. Consequently, replication-dependent histone gene expression is coordinated with DNA synthesis. 

#### 2.7.5. Human Diseases Associated with the Histone Locus Body

No human diseases have been described for mutations in genes encoding for proteins or RNAs that comprise histone locus bodies.

### 2.8. Paraspeckle

#### 2.8.1. Discovery

Paraspeckles are a recently discovered nuclear body found through a proteomic study of purified human nucleoli [12]. This analysis identified 271 nucleolar proteins, 80 of which were encoded by novel or uncharacterized human genes [12]. Further characterization of non-POU (Pit1/Oct1/UNC-86) domain containing octamer-binding (NONO), paraspeckle protein 1 (PSP1) and paraspeckle protein 2 (PSP2) detected a nuclear compartment located close to, but distinct from, nuclear speckles [78]. 

#### 2.8.2. Key Components of the Paraspeckle

Established paraspeckle component proteins include the aforementioned NONO, PSP1 and PSP2, as well as splicing factor proline/glutamine-rich (SFPQ) [280] and mammalian cleavage factor I 68 (CFIm68) [80]. Additionally, a recent study identified 35 additional paraspeckle proteins, including RNA-binding proteins, additional members of the CFIm complex and several hnRNPs that bind RNA Pol II transcripts [281]. Paraspeckles also contain the long ncRNA nuclear-enriched abundant transcript 1 (*NEAT1*) [81,82,83] and nuclear-retained RNA CAT2 transcribed nuclear (*Ctn*) [79] (Table 1).

#### 2.8.3. Functions of Key Components

Paraspeckle proteins participate in several biological processes. PSP1, NONO and SFPQ are members of the *Drosophila* behavior/human splicing (DBHS) family of RNA-binding proteins that possess two tandem RNA recognition motifs (RRMs). The DBHS family proteins are involved in nuclear processes, such as transcription, pre-mRNA processing and DNA repair [129,282,283,284,285,286,287,288,289,290]. RNAi-mediated knockdown of either *NONO* or *SFPQ* leads to a loss of paraspeckles [83]. Animal models targeting DBHS homologues have diverse defects, including those of circadian rhythm, chondrogenesis and neural development [129,130,291] (Table 2). 

PSP2 mediates transcription and splicing in a promoter-preferential manner [292]. CFIm68, a subunit of the multimeric CFIm complex, facilitates the recognition of pre-mRNA and the recruitment of factors for pre-mRNA 3'-end processing [293], as well as for nuclear export of mRNA [294]. 

The *NEAT1* RNA is a mammal-specific ncRNA that is developmentally regulated. It is widely expressed in adult mouse tissues, but not earlier in development [133]. Studies of *NEAT1* knockdown in cultured human cells and of a knockout *Neat1* mouse show that these RNAs are essential for paraspeckle formation [81,82,83,133] (Table 2); however, the *Neat1* RNA is not needed for mouse viability, health or fertility [133] (Table 2). 

The mouse-specific *Slc7a2* gene encodes two isoforms: the nuclear retained *Ctn* RNA and the protein coding *Cat2* mRNA [79]. The nuclear retention of *Ctn* RNA is mediated by the post-transcriptional modification of 3' UTR adenosine to inosine (also known as A-to-I editing) and subsequent binding of the modified RNAs to the paraspeckle components, NONO and SFPQ, as well as matrin 3 [79,295]. In contrast to *NEAT1* RNA, depletion of *Ctn* RNA by antisense oligonucleotide (ASO) knockdown has no effect on paraspeckle formation [79] (Table 2).

#### 2.8.4. Mechanisms of Gene Expression and the Paraspeckle

The precise biological role of paraspeckles is unknown; however, analysis of the function of the individual components suggests that paraspeckles contribute to transcriptional regulation and RNA processing (Table 1, Table 2). Since several paraspeckle components are involved in transcription and RNA processing, the paraspeckle might contribute to the coupling of these events, such that the cell can streamline consecutive enzymatic reactions [129,282,283,286]. 

A function particular to paraspeckles is nuclear RNA retention [79,295]. Exemplifying this function is *Ctn* RNA [79]. While nuclear *Ctn* RNA has the same coding exons as its protein-coding cytoplasmic counterpart, *Cat2* mRNA, *Cat2* mRNA is transcribed from an alternative promoter and utilizes a distal poly(A) site, resulting in a longer 3' UTR [79]. Following stress, *Ctn* RNA is cleaved at its 3' UTR, exported from the nucleus and translated into the Cat2 protein [79]. Cat2 is an amino acid transporter required for the uptake of arginine. Arginine is required for nitric oxide synthesis by the L-arginine-nitric oxide pathway, which is induced by stresses, such as infection and wound healing [296]. Nuclear RNA retention, therefore, provides an accessible pool of ready-to-use transcripts for rapid responses to stimuli. Although only a single mouse-specific RNA is known to be regulated by nuclear RNA retention [79], it is likely that this mechanism also exists in humans, since many human poly(A)+ RNAs are retained in the nucleus [297], and numerous A-to-I edited human RNAs have been identified [298,299,300]. 

#### 2.8.5. Human Diseases Associated with the Paraspeckle

No human diseases have been described for mutations in genes encoding for paraspeckle proteins or RNAs. 

## 3. Discussion

As proposed by Rabl in 1885 [301], interphase chromosomes have a territorial organization and subsequent studies have shown that the DNA from chromosomes is not randomly intertwined, but rather, occupies non-overlapping territories of irregular shape [302,303,304,305,306]. Genes are generally distributed along the periphery of chromosome territories and loop out into interchromosomal domains upon the induction of gene expression [307,308,309,310,311,312,313,314,315,316,317]. Nuclear bodies, such as Cajal bodies and nuclear speckles, and specific nascent RNA accumulations lie in the interchromosomal domains and are excluded from the chromosome territories [318,319,320,321,322]. Given these observations and the finding that the positioning of interphase chromosomes is frequently inherited from mother to daughter nuclei in mammals, the spatial relationship of DNA to nuclear bodies may represent a higher order mechanism for regulating gene expression [323,324,325,326,327]. Consistent with such a hypothesis, chromosomal translocations cause large-scale changes in gene expression attributable to the change in the chromosome territory [328]. Aside from differences in interaction with the nuclear matrix, one contributor to this change in gene expression could be the change in the spatial relationship with the nuclear compartments that concentrate and sequester factors required for gene expression or chromatin homeostasis. 

Besides potentially regulating gene expression based on concentration or sequestration of factors, nuclear bodies provide a structure for cells to couple and integrate sequential processes in order to increase the efficiency and tuning of gene expression. Although our understanding of this integration is primitive, traces of this integration can be observed in the way in which nuclear bodies share factors and interact with other nuclear bodies and nuclear components in time and space. Many factors are not exclusive to one subnuclear domain and have functions in several domains, e.g., coilin and the SMN complex. This sharing of factors not only allows the cell to maximize the function for any given protein, but also potentially provides a mechanism by which different nuclear bodies can communicate with one another. For instance, the transfer of SMN between the Cajal body and the Gemini of Cajal body might be a mechanism of communication and of modulating their respective functions. Finally, the histone locus body and its role in the transcription of the replication-dependent histone genes and the seamless coordination with the cell cycle exemplify the interaction of a nuclear body with temporal nuclear functions. 

Several observations also suggest that this integration extends beyond the modulation of gene expression to that of a fundamental role in global nuclear homeostasis. Many recent studies have uncovered intertwining of the processes of DNA transcription, replication, repair and recombination. Although partially attributable to the common substrate DNA and some proteins functioning in multiple processes, nuclear bodies frequently act as nexi contributing to each or many of these processes [9,10,43,44,69,99,129,196,233,329,330,331,332,333,334,335].

As might be predicted for structures mediating responses to stimuli, nuclear bodies are highly dynamic [336,337]. They rapidly assemble or disassemble, as well as change location upon exposure of a cell to various stimuli. Examples of such dynamic responses include the formation of nucleolar caps upon transcription inhibition [338], the formation of nuclear stress bodies in response to cellular stress [40,201], the altered distribution and enlargement of nuclear speckles upon transcription inhibition or heat shock [191,339] and the rapid reassembly of several nuclear bodies following mitosis [337]. Further examples include the increased association between Cajal bodies and Gemini of Cajal bodies, the appearance of paraspeckles and the marked changes in nucleolar positioning, number and association with chromatin territories upon cellular differentiation [131,340,341]. 

Reinforcing the vital nature of nuclear bodies, they are conserved across species that have been well studied (Table 4). However, very few studies have looked at conservation of the position of nuclear bodies relative to genes across species. One example is the relationship between nucleoli and nucleolar organizer regions (NORs) in nuclei of pachytene spermatocytes from seven mammalian species [342]. NORs consist of tandemly repeated ribosomal genes around which nucleoli form [343,344]. The localization of the nucleoli was largely dependent upon the position of the NOR relative to the chromosome: terminal NORs invariably gave rise to peripheral nucleoli, while intercalated NORs gave rise to central nucleoli [342]. The number of nucleoli was largely dependent on the number of nucleolar bivalents: a single nucleolar bivalent gave rise to a single nucleolus, while multiple nucleolar bivalents seeded a variable number of nucleoli [342]. Another comprehensive study of the localization of ribosomal genes in 189 species of animals and plants with diverse karyotypes showed that 90.5% of NORs were located on a short arm and 85.2% near the telomere [345,346]. Based on these initial studies, one might hypothesize that nucleoli are non-randomly distributed nuclear bodies with species-specific variation in nuclear architecture. Selection for these interactions would be predicted to have a stabilizing effect on the structure of the genome and the three-dimensional structure of the nucleus. Another nuclear body where position relative to genes might be conserved is the histone locus body. The core replication-dependent histone genes show high conservation across species [347], and it has been demonstrated that histone H2B pre-mRNA is sufficient to nucleate histone locus bodies *de novo* [235]. In agreement with this finding, it has recently been shown that a sequence located between the *Drosophila melanogaster* histone H3 and H4 genes, as well as the transcription of the histone H3 and H4 genes facilitate histone locus body formation [128]. Since selection for this interaction would be necessary for the survival of the cell, other species are likely to have a similar mechanism of histone locus body nucleation.

Further work on these intriguing aspects of nuclear bodies will no doubt provide more knowledge regarding the function and regulation of nuclear bodies, as well as their contribution to the regulation of gene expression.

## 4. Conclusion

Nuclear bodies utilize a vast array of gene regulatory mechanisms for efficient and controlled gene expression (Figure 1). Further work to uncover the biological functions, interactions and dynamics of nuclear bodies will give us greater insight into the organizational architecture and three-dimensional landscape of the nucleus and the spatial regulation of gene expression within the nucleus. Although nuclear bodies have profound effects on gene regulation and gene expression, they have been generally underappreciated with respect to their function in health and disease. This emphasizes the need to understand disease in the spatial context of the nucleus. 

The study of nuclear bodies and the mechanisms by which they regulate gene expression is an exciting, but complex and challenging area of cell biology. There are likely more nuclear bodies, as well as components and functions of existing nuclear bodies to be discovered and elucidated. 
